# Isolation and Characterization of Marine *Brevibacillus* sp. S-1 Collected from South China Sea and a Novel Antitumor Peptide Produced by the Strain

**DOI:** 10.1371/journal.pone.0111270

**Published:** 2014-11-05

**Authors:** Lanhong Zheng, Yao Yi, Jia Liu, Xiukun Lin, Kangli Yang, Mei Lv, Xinwen Zhou, Jianhua Hao, Junzhong Liu, Yuan Zheng, Mi Sun

**Affiliations:** 1 Key Laboratory of Sustainable Development of Marine Fisheries, Ministry of Agriculture, Yellow Sea Fisheries Research Institute, Chinese Academy of Fishery Sciences, Qingdao, PR China; 2 Qingdao University, Qingdao, PR China; 3 Department of Pharmacology, Capital Medical University, Beijing, PR China; 4 Qingdao University of Science & Technology, Qingdao, PR China; 5 Institutes of Biomedical Sciences, Fudan University, Shanghai, PR China; CAS, China

## Abstract

A Gram-positive, rod-shaped bacterium, designated as S-1, was isolated from a marine sediment sample collected from South China Sea. Phylogenetic analysis based on 16S rRNA gene sequence showed that S-1 belongs to the genus *Brevibacillus*. A novel cytotoxic peptide was isolated from the fermentation broth of the marine-derived bacterium *Brevibacillus sp.* S-1, using ion-exchange chromatography and reverse-phase HPLC chromatography. The molecular weight of this peptide was determined as 1570 Da by MALDI-TOF mass spectrometry, and its structure was proposed as a cyclic peptide elucidated by MALDI-TOF/TOF mass spectrometry and *de novo* sequencing. 3-(4,5-dimethyl-2-thiazolyl)-2,5-diphenyl-2H-tetrazolium bromide (MTT) assay showed that this peptide exhibited cytotoxicity against BEL-7402 human hepatocellular carcinoma cells, RKO human colon carcinoma cells, A549 human lung carcinoma cells, U251 human glioma cells and MCF-7 human breast carcinoma cells. Additionally, SBP exhibited low cytotoxicity against HFL1 human normal fibroblast lung cells. The result suggested that the cytotoxic effect of the peptide is specific to tumor cells.

## Introduction

As the leading cause of death, malignant tumors seriously threaten human health. The incidence of cancer keeps growing in recent years. About 12.7 million cancer cases and 7.6 million cancer deaths occurred in 2008 on the basis of GLOBOCAN 2008 [1]. Similar to surgical resection, radiotherapy, and immunotherapy, chemotherapy is widely used for clinical treatment of malignancy. Although the current drugs have certain curative effects on some types of cancers, many problems, such as poor specificity, low antitumor activity and great side-effects, greatly limited their clinical application [2]. Therefore, the identification of new drugs with high efficiency, low toxicity and a great specificity is an urgent task in the field of cancer research.

In addition to plants and animals, microorganisms are an alternative major resource for the discovery of new drugs. More than 50,000 microbial natural products have been obtained during the history of drug discovery. The majority of these compounds are isolated from terrestrial microbes [3]. However, after 50 years of intensive screening from terrestrial microbes, the pace of discovery and development of microbial natural products with a unique scaffold has dramatically decelerated over the last two decades. Recent trends in drug discovery emphasize that marine microorganisms are a potentially productive resource of novel secondary metabolites and There is a great potential to increase the number of marine natural products in clinical trials [4]. In contrast to the terrestrial environment, the oceans are a rich and relatively untapped reservoir of novel nature products. Over 15,000 structurally diverse natural products with many bioactivities have been identified from marine environments since the 1970s [5]. Marine microbial natural products have attracted increasing attention from microbiologists, taxonomists, ecologists, chemists and evolutionary biologists during a few recent decades. The search for marine microbial natural products has just begun, However, over 30 compounds derived from marine microbes, such as Didemnin B (Aplidine) and thiocoraline, are undergoing clinical or preclinical studies for the treatment of different types of cancers [6], [7]. Numerous studies have indicated that diverse marine microbes appear to have the capacity to produce an impressive array of marine microbial natural products exhibiting a wide range of biological activities, such as anti-tumor, antimicrobial and anti-inflammatory activities. Marine microorganisms represent an unexplored reservoir for the discovery of marine microbial natural products with unique scaffolds and for exploitation in the pharmaceutical industries [8].

The number of peptides with antitumor activity discovered from marine organisms has a growing trend. Recent advances on anticancer peptides from marine resource have provided novel information about marine bioactive peptides [9]. These findings have contributed to our understanding of the relationship between chemical structure of peptides and biological activity [10]. These facts demonstrate marine peptides as a new source of obtaining lead compounds for biomedical purposes.

## Materials and Methods

### Reagents

HiPrep CM FF 16/10 column was purchased from GE Healthcare. SunFire C18 column (19×150 mm) was purchased from Waters. Penicillin-Steptomycin, MTT, dimethyl sulfoxide (DMSO), Diethylpyrocarbonate (DEPC) was obtained from Sigma Chemical Co. (St. Louis, MO, USA). Fetal calf serum and Dulbecco's Modified Eagle's Medium (DMEM) culture medium supplemented with Glutamine were purchased from Gibco Invitrogen (Carlsbad, CA, USA). Bicinchoninic acid (BCA) protein assay kit was obtained from Thermo scientific (Pierce Inc., Rockford, IL, USA). Other commercially available chemicals and reagents were analytical grade. All reagents were prepared using deionized MilliQ water.

### Isolation of marine microorganisms

No specific permissions were required for the location. The field studies did not involve endangered or protected species. The sea sediment was collected from the bottom of northern South China Sea (Station E201: North latitude 21°41′, East longitude 116°18′), stored in the refrigerator, and brought to the laboratory. The microorganism strains were isolated by plated 200 µL different diluted sea sediment (from 10^−1^ to 10^−7^) on beef extract-peptone medium agar plates in triplicates. The inoculated plates were incubated at 25°C, respectively for 2–3 days. Colonies arising on all solid plates were selected based on their physiological features and morphological characteristic including rate of growth, shape, size pigmentation and margin. Among 96 bacterial strains isolated one strain (named S-1) that showed the strongest cytotoxic effect on BEL-7402 cell lines by MTT assay was chosen for further studies. The selected strains were treated with streak plate to check their purity and conserved in15–20% glycerol at −80°C.

### Morphological, physiological and biochemical characteristics of strain S-1

Standard protocols [11] were used to assess oxidase activities, degradation of gelatin, nitrate reduction, carbon source utilization and H_2_S production from thiosulfate [12].

### Phylogenetic analysis

Chromosomal DNA of the strain S-1 was extracted [13] and the 16S rRNA gene was amplified by PCR using universal primers 27f 5′-AGAGTTTGATCCTGGTCAG-3′; and 1492r 5′-CGGCTACCTTGTTACGAC-3′ [14]. Purified PCR products were ligated into the pMD 18-T (TaKaRa) according to the manufacturer's instructions. Sequencing reactions were carried out using ABI BigDye 3.1 Sequencing kits (Applied BioSystems) and an automated DNA sequencer (model ABI3730; Applied BioSystems). The near-complete 16S rRNA gene sequence of strain S-1 was submitted to GenBank/EMBL to search for similar sequences using the BLAST algorithm. The identification of phylogenetic neighbours and the calculation of pairwise 16S rRNA gene sequence similarities were achieved using the EzTaxon server (http://www.eztaxon.org/) [15]. Sequences were aligned using CLUSTAL X1.8 [16]. Phylogenetic trees were constructed using the 5eighbor-joining methods implemented in the program MEGA version 6 [17]. In each case, bootstrap values were calculated based on 1000 replicates.

### Inoculum and fermentation

The basal medium used for antitumor production consisted of 1.0% tryptone, 0.3% beef extract and 0.5% NaCl (pH 6.5∼7.0). Incubation was carried out at 25–30°C in a rotary shaker, with stirring at 180–200 rpm. A loopful of cells from a slant was transferred to 25 mL of the above mentioned sterile medium in a 250 mL Erlenmeyer flask and incubated at 30°C and 200 rpm for 16 h. This was used as the inoculum. Fermentation was carried out in 1000 mL Erlenmeyer flasks, each containing 200 mL of the sterile production medium. The medium was inoculated with 4% (v/v) of 16 h old culture. The inoculated flasks were kept on a rotary shaker at 25–30°C and 180–200 rpm for 36–48 h.

### Preparation of crude extract

For the production of secondary metabolites, the marine bacterial isolate *Brevibacillus sp.* S-1 was cultured as above. After centrifugation (10000 r/min, 15 min), the supernatant was collected and then was fractionated with a separating funnel an equal volume of n-butyl alcohol was added and vigorously shaken for 5 min. The organic (upper) layer was carefully separated and an equal volume of fresh n-butyl alcohol was added and the extraction into the organic layer was repeated twice. The pooled organic layer was evaporated to dryness in a rota evaporator under reduced pressure (Buchi, Germany). The extract pellet was dissolved in Glycine and sodium hydroxide buffer (50 mmol/L, pH 9.0) for further usage.

### Purification of peptide from the strain S-1

#### Cation- exchange chromatography

The peptide pellet was dissolved in Glycine and sodium hydroxide buffer (50 mmol/L, pH 9.0) and loaded onto a HiPrep CM FF 16/10 column chromatography which had been previously been equilibrated with the above buffer. A stepwise elution eluted with was dialyzed against the Glycine and sodium hydroxide buffer (50 mmol/L, pH 9.0), washed with the same buffer to remove unabsorbed proteins. The adsorbed proteins were eluted with 0–25% containing 1 mol/L NaCl prepared in the same buffer at a flow rate of 5 mL/min. Each fraction was collected at a volume of 50 mL and was monitored at 280 nm. All of the fractions were desalted by dialyzing against ultra-pure water and antitumor activities were determined. The fraction having the strongest cytotoxicity activity was collected and used for further experiments.

#### Reversed phase-high performance liquid chromatography (RP-HPLC)

HPLC analyses on a Waters 2545-2767-2489 HPLC system fitted with a WATERS SunFire C18 column, 19×150 mm. the elution solvent system was composed of water- trifluoroacetic acid (TFA) (solvent A, 100∶0.1, v/v) and acetonitrile (ACN)-TFA (Solvent B, 100∶0.1, v/v). The peptide was further purified using a gradient elution 5% of solvent B in 5 min, 33% of solvent B in 5 min, 38% of solvent B in 35 min, 100% of solvent B in 40 min at a flow rate of 3 mL/min. UV detection was set at 254 nm and column temperature was 24°C.

### The determination of peptide concentration

Protein concentrations were determined using the BCA protein assay kit and Bovine serum albumin (BSA) as the standard.

### Molecular mass determination and identification of peptide by MALDI-TOF/TOF-MS

To determine the precise molecular weight of the purified peptide was using MALDI-TOF. The amino acid sequence of peptide was identified by the reference with some modifications [18]. An amount of 0.5 µL of the sample was dissolved in water of HPLC grade and spotted onto MALDI sample target plate, and then 0.5 µL of 0.5 g/L α-cyano-4-hydroxycinnamic acid (CHCA) prepared in 50% CAN/0.1% TFA was added. Peptide mass spectra were obtained on a 5800 MALDI-TOF/TOF (Applied Biosystems, SCIEX, Foster City, CA, USA) instrument equipped with a 355 nm nitrogen laser for desoption and ionization, as described previously [19]. After an external calibration with a mixture of enzymatic hydrolysis peptides about myoglobin, spectra were obtained in the mass range between 700–3600 Da. For each sample spot, a data dependent acquisition method was created to select the most intense peaks for subsequent MS/MS data acquisition. To ensure a reliable identification, the results from both the MS and MS/MS spectra were used in the database search. Peptide identification was accepted when the score read by the Mascot search routine was higher than 90. The sequence of peptide fragments was determined by *de novo* sequencing using the Applied Biosystems software as presented by Yergey [20].

### Cell culture

BEL-7402 human hepatocellular carcinoma cells, RKO human colon carcinoma cells, A549 human lung carcinoma cells, U251 human glioma cells, MCF-7 human breast carcinoma cells, and HFL1 human normal fibroblast lung cells were provided by the Cell Bank of Chinese Academy of Sciences (Shanghai, China). BEL-7402, RKO, A549, U251 and MCF-7 cells were cultured in DMEM supplemented with 10% heat-inactivated fetal bovine serum, 100 U/mL of penicillin and 100 mg/mL streptomycin. HFL1 cells were cultured in F12K supplemented with 10% heat-inactivated fetal bovine serum, 100 U/mL of penicillin and 100 mg/mL streptomycin. Cells were grown in a humidified atmosphere containing 5% CO_2_ at 37°C.

### The assessment of cytotoxicity

The inhibitory effects of the peptide from strain S-1 on the viability of BEL-7402 cells, RKO cells, A549 cells, U251 cells and MCF-7 cells and normal HFL1 cells were evaluated by MTT assay [21]. Some improvement was made in our study. In brief, the above cancer cells (4×10^3^) in 180 µL of DMEM culture media were seeded into each well on 96-well microplates and cultured for 24 h. Then, 20 µL the peptide with certain concentrations were added to the media. After incubation for 48 h, MTT solution (20 µL, 0.5 mg/mL) was added to each well, and the cells were cultured for another 4 h at 37°C. The media were removed and 150 µL DMSO was added to dissolve the formazan crystals. The OD_570_ was measured by an Infinite M200 PRO microplate reader (TECAN Group Ltd, Mannerdorf, Switzerland) with subtraction of background absorbance. All experiments were performed in triplicate. The cytotoxicity of the peptide was expressed as an IC_50_ value, defined as the concentration causing a 50% reduction of cell viability compared with untreated cells. The percentage of cytotoxicity was calculated as follows: Relative inhibition rate (%)  =  [(A_570_ value of the control-A_570_ value of the experimental samples)/A_570_ value of the control] ×100%.

### Statistical analysis

All of the tests were conducted in triplicate, and the experimental data were expressed as the mean ± SD. The statistical significance of the mean difference between the control and treated groups was determined by a paired t-test; p<0.05 was considered statistically significant.

## Results and Discussion

### Phenotypic and phylogenetic characterization of the strain S-1

**Figure 1:**
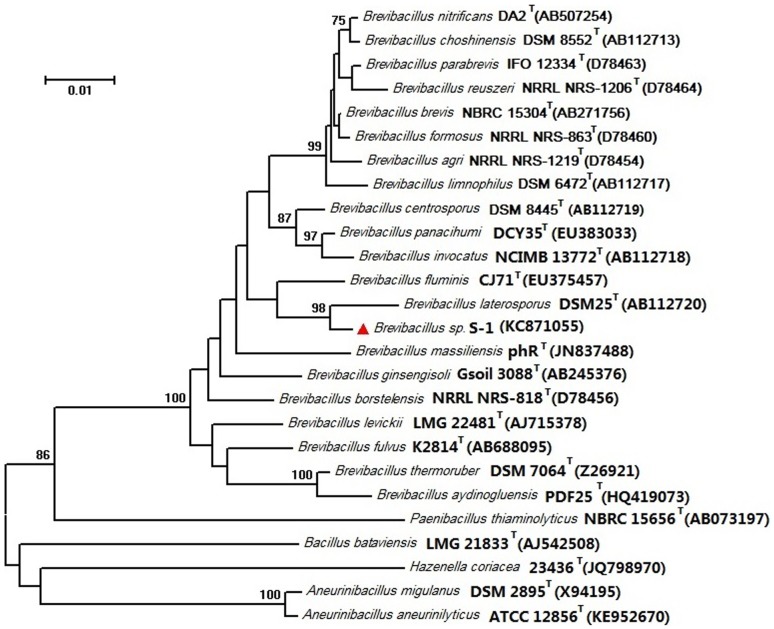
Phylogenetic dendrogram of *Brevibacillus sp.* S-1 and its related species based on 16S rRNA gene sequence similarities. The tree was constructed using the neighbour-joining method implemented in the program MEGA version 6. Bar, 0.01 nt substitutions per site.

Marine microorganisms are a major source for natural products. Now, 16 of 20 marine antitumor compounds under clinical trial are derived from microbial sources [22]. Therefore, isolation and cultivation of a new marine microorganism may be a shortcut to discover novel natural products [23]. A variety of pretreatment methods including enriching physical and chemical techniques are employed to favor the isolation of specific marine microorganisms, especially less abundant bacteria. [24], [25]. Anticancer ε -poly-L-lysine (ε -PL) was produced by a marine *Bacillus subtilis* sp. isolated from sea water in Alexandria [26]. We isolated a strain, S-1, from the bottom sediments of South China Sea. The near-complete 16S rRNA gene sequence (1466 nt) of strain S-1 was submitted to GeneBank (GenBank accession number KC871055). Phylogenetic analysis based on the 16S rRNA sequence indicated that strain S-1 belonged to the genus *Brevibacillus*, with the highest sequence similarities to *Brevibacillus laterosporus* DSM25 (98.29%), *Brevibacillus panacihumi* DCY35 (97.26%), *Brevibacillus invocatus* NCIMB 13772 (96.99%), *Brevibacillus fluminis* CJ71 (96.89%) and *Brevibacillus centrosporus* DSM 8445 (96.78%). The neighbour-joining phylogenetic tree further confirmed that strain S-1 was phylogenetically related to genus *Brevibacillus* and formed a clade with *Brevibacillus laterosporus* (Figure 1).

In addition to the 16s rRNA sequence that defined the genus, the following characteristics are also observed. *Brevibacillus sp.* S-1 is Gram-positive and rod-shaped. The colonies are uniformly round, regular, convex, smooth and 0.1–1.0 mm in diameter. The ranges of temperature, pH, and NaCl concentration for S-1 growth are 15.0–37.0°C, 5.0–9.0, and 2.0–7.0% (w/v), respectively. Its oxidase reaction and nitrate reduction reaction are both positive, while the Voges-Proskauer (V-P) reaction is negative. Gelatin is hydrolysed by S-1, while citrate cannot be utilized. S-1 produces neither H_2_S nor indole. Acid can be produced from D-glucose and D-mannitol, but not L-arabinose, by S-1. No gas was obtained from D-glucose-treated S-1 strain.

### The fermentation and preparation of crude extract

Fermentation was carried out in 1000 mL Erlenmeyer flasks, each containing 200 mL of the sterile production media. For the production of secondary metabolites, the marine *Brevibacillus* sp. S-1 was cultured as described in Section of Materials and methods. About 50 L of production media was prepared. After centrifugation, the supernatant was collected and then was fractionated with n-butyl alcohol. The pooled organic layer was evaporated to dryness using a rotary evaporator under reduced pressure. The extract pellet was dissolved in glycine-sodium hydroxide buffer (50 mmol/L, pH 9.0) for further usages.

### Purification of novel antitumor peptides from *Brevibacillus* sp. S-1

**Figure 2:**
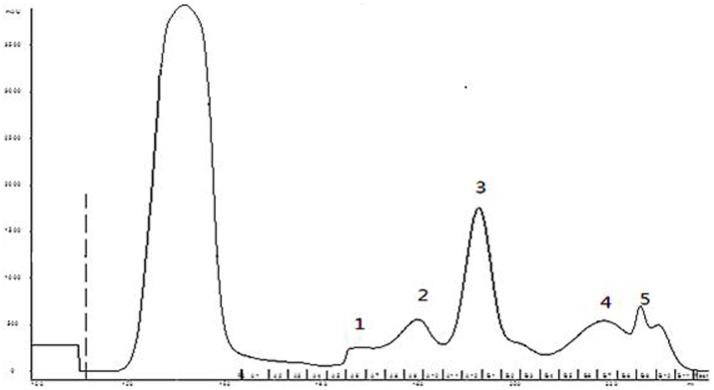
Separation profile of the crude extract from fermentation products of *Brevibacillus* sp. S-1 on a CM-Sepharose Fast Flow.

**Figure 3:**
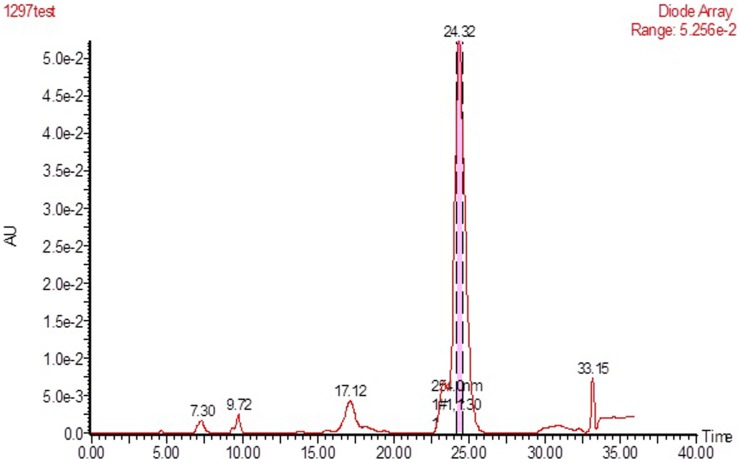
Reversed phase-high performance liquid chromatography (RP-HPLC) profile of SBP performed on a Waters 2545-2767-2489 HPLC system fitted with a Waters SunFire C18 column, 19×150 mm.

We developed a purification protocol involving cation-exchange chromatography and reversed phase-high performance liquid chromatography (RP-HPLC). The crude peptides were dissolved with the glycine-sodium hydroxide buffer (50 mmol/L, pH 9.0), and the dissolved solution was applied to CM sepharose Fast Flow column, washed with the same buffer to remove unattached peptides and proteins. The attached peptides and proteins were eluted with linear gradient elution from 0% to 25% of 1 M sodium chloride (NaCl) solution in the same buffer (Figure 2). All of the fractions were desalted by dialyzing against ultra-pure water. The resulting solution was then prepared as freeze-dried powder, and was re-dissolved when used. The cytotoxicity of each fraction to BEL-7402 cells was determined by MTT assay. Fraction 3, which was identified to be cytotoxic to tested cancer cells, was submitted to reverse phase chromatography using a C18 column, and purified for homogeneity (Figure 3). We named this antitumor peptide from *Brevibacillus sp.* S-1 as SBP. The yield is about 0.25 mg/L. To achieve a higher production, fermentation conditions and culture medium need to be further optimized.

### Molecular mass determination and *de novo* sequencing of SBP

**Figure 4:**
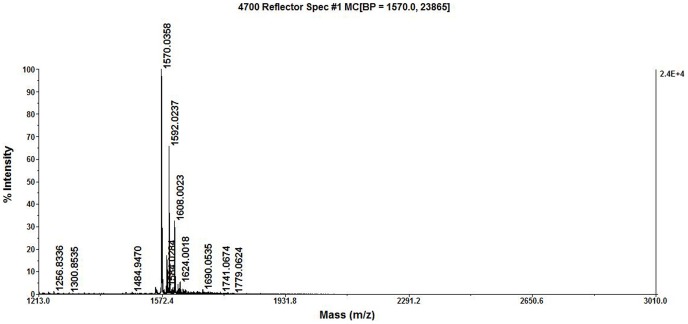
The molecular ion peak of SBP [M+H]^+1^ to 1570.0358 measured by MALDI TOF/TOF Mass spectrum.

**Figure 5:**
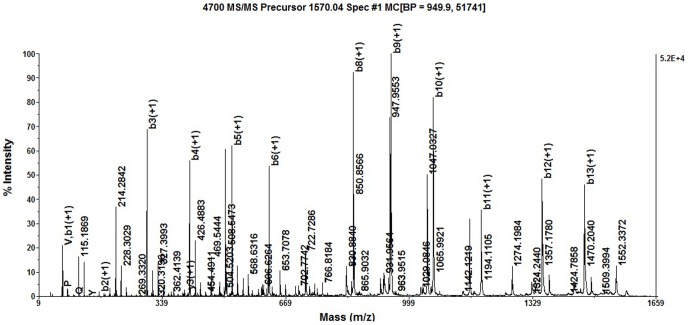
Analysis of MALDI TOF/TOF MS/MS about the molecular ion peak of 1570.0358.

Matrix Assisted Laser Desorption Ionization Time of Flight Mass Spectrometry (MALDI TOF/TOF MS) analysis of SBP was performed with different ion signals in the mass range of 700–3600 Da. 1570.0358, 1592.0237 and 1608.2003 is M+H, M+Na and M+K ions, respectively. The molecular weight of SBP was about 1570 Da, as determined by MALDI TOF/TOF MS analysis (Figure 4). The precursor ions (m/z 1570. 0358) were further detected by MS/MS analysis. MS/MS spectra consisting of a series of y and b ions were obtained and used for *de novo* sequencing. Based on the manual calculation of the molecular weights and the m/z values, the amino acid sequence of SBP peptide was proposed. The amino acid sequence of fragment ion m/z 1570.0358 was the APQNI/LVPKTI/LKYI/LC and the peptide was structurally cycle (Figure 5). One of the amino acids in this sequence is Thr, and therefore, SBP may be a cyclic peptide linked end-to-end, or a cyclic depsipeptide linked with a Thr-(-OH)-to-Cys(-COOH) bond. However, the possibility of being a cyclic desipeptide is quite low because of bigger space steric hindrance between Thr and Cys. The theoretical M+H ion of the proposed cyclic peptide is 1569.925 Da, and in agreement with the exact mass observed for the M+H ion (figure 4). The molecular weight of Ile and Leu is same, so these two amino acids could not be determined according to available data. The results of the present study indicated that the amino acid sequence of SBP was the APQNI/LVPKTI/LKYI/LC, and the SBP was most likely to be a cyclic peptide linked in an end-to-end fashion. The amino acid sequence alignment was performed against the National Center for Biotechnology Information Basic Local Alignment Search Tool (NCBI BLAST) database, and the low similarity of its amino acid sequences with the known proteins indicated that SBP was a novel peptide.

### The cytotoxicity of SBP against tumor cells

**Figure 6:**
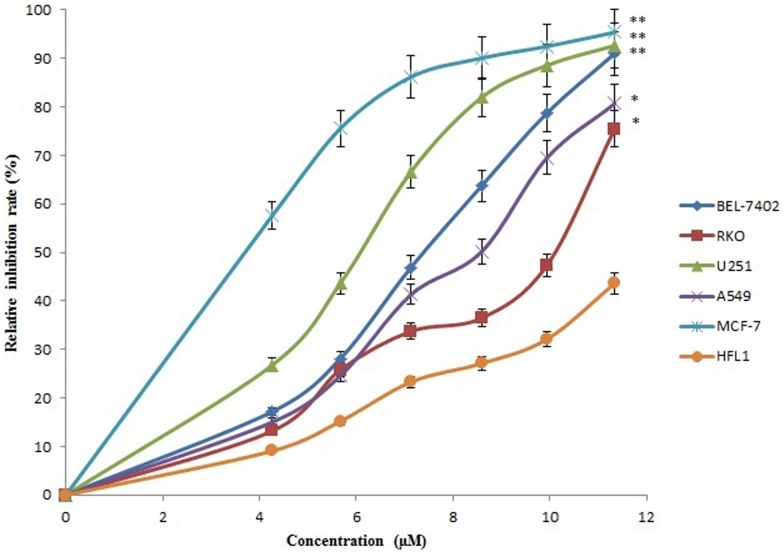
Cytotoxicity of SBP to tumor cells. BEL-7402 Human hepatocellular carcinoma cells, RKO human colon carcinoma cells, A549 human lung carcinoma cells, U251 human glioma cells, MCF-7 human breast carcinoma cells, and HFL1 human normal fibroblast lung cells were treated with certain concentrations of SBP for 48 h. The cell inhibitory rate was determined by MTT assay as described in experimental section. Data were presented as means ± SD of three independent experiments; *p<0.05, compared with HFL1 human normal fibroblast lung cells.

The cytotoxicity of SBP was determined using MTT assay. As shown in Figure 6, SBP significantly inhibited the viability of the selected five cancer cell lines in a dose-dependent manner. The IC_50_ values were 7.15, 10.45, 8.41, 6.49, and 3.38 µM for BEL-7402 human hepatocellular carcinoma cells, RKO human colon carcinoma cells, A549 human lung carcinoma cells, U251 human glioma cells, and MCF-7 human breast carcinoma cells after treatment for 48 h, respectively. Additionally, SBP displayed low cytotoxicity against HFL1 human normal fibroblast lung cells. The result suggested that the inhibitory effects of the peptide on cell viability exhibited some specificity to tumor cells.

Some cyclic peptides have been discovered from marine microorganisms with potent antitumor activities in clinical trial. Aplidine (dehydrodidemnin B, Aplidin), a cyclic depsipeptide, has been isolated from the Mediterranean tunicate *Aplidium albicans*. The data revealed that breast, melanoma, and non-small-cell lung cancer appear to be sensitive to low concentrations of Aplidine [27], [28]. Aplidin has entered into phase II clinical trials in Europe and Canada for the treatment of renal, head and neck, and medullary thyroid tumors [29]. Didemnin B, a branched *N*-methylated cyclic peptolide, was originally isolated from the *Trididemnum* genus of marine tunicates. This peptide induces the death of a variety of transformed cells, evidenced by nucleic shrinking, DNA fragmentation, and the generation of DNA ladders [30]. Didemnin B is the first marine peptide that has been entered into human clinical trials in the USA for the treatment of cancer, and has completed phase II human clinical trials for the therapy of kidney adenocarcinoma [31], advanced epithelial ovarian cancer [32], and metastatic breast cancer [33]. The screening and development of optimal culture media for peptide SBP production from *Brevibacillus sp.* S-1 are in progress in our laboratory. The fermentation cultivation can meet the demand of medical proposes. It is important that SBP is water-soluble peptide with small molecular weight, and has high thermal stability. These advantages are beneficial for its conservation and medicinal usage. SBP is derived from secondary metabolites of *Brevibacillus* sp. S-1 isolated from the special marine environment, and maybe has its own special targets in cancer cells, rather than in normal cells. So the molecular mechanism of SBP to kill cancer cells should be an important issue in the further research. At the same time, the *in vivo* anticancer potential activity of SBP should be investigated in the future.

## Conclusions

In this study, the strain *Brevibacillus sp.* S-1 was isolated from a marine sediment sample collected from South China Sea. Furthermore, we extracted and purified a novel cyclic peptide, SBP, with a MW of 1570 Da from the fermentation products of *Brevibacillus sp.* S-1. MTT assay indicated that SBP exerted a promising antitumor activity on cancer cell lines.
