# Correction: Isolation and Characterization of Marine *Brevibacillus* sp. S-1 Collected from South China Sea and a Novel Antitumor Peptide Produced by the Strain

**DOI:** 10.1371/journal.pone.0114974

**Published:** 2014-12-02

**Authors:** The PLOS ONE Staff

In the Funding section, the grant numbers from the funder Chinese Academy of Fishery Sciences and the funder Science and Technology Development Plans of Shandong Province are listed incorrectly. The correct grant numbers are 2014B01YQ01 and 2014GSF121016, respectively.
